# The protective effect of Saudi Arabian bee honey against excessive weight gain and obesity-related parameters in rats fed a high-fat diet

**DOI:** 10.3389/fnut.2025.1582408

**Published:** 2025-08-18

**Authors:** Abdullah Al Tamim, Ghedeir M. Alshammari, Abu ElGasim A. Yagoub, Ali Saleh, Mohammed A. Mohammed, Mohammed Abdo Yahya

**Affiliations:** Department of Food Sciences and Nutrition, College of Food and Agricultural Sciences, King Saud University, Riyadh, Saudi Arabia

**Keywords:** Talh honey, Sidr honey, obesity, high-fat diet, AMPK, Nrf2, oxidative stress, inflammation

## Abstract

**Introduction:**

This study aimed to investigate the anti-obesity, hepatic protective, and metabolic effects of Sidr and Talh honey, two Saudi honey, in rats fed a high-fat diet (HFD) and examined some possible mechanisms of their action.

**Methods:**

Adult rats were divided into eight groups (*n* = 8 each) and were administered HFD for 12 weeks, with or without oral doses of Sidr or Talh honey at 500, 700, and 1,000 mg/kg.

**Results and Discussion:**

Talh honey significantly reduced body weight, fat mass, and adiposity markers, including mesenteric, subcutaneous, and epididymal fat, compared to the HFD group. It also improved plasma glucose, insulin, HOMA-IR, HbA1C, leptin, triglycerides, cholesterol, LDL-c, and increased adiponectin. Sidr honey showed no effects on the majority of these factors, except it was able to lower glucose, HbA1C, and HOMA-IR, but was less effective than Talh honey. Both honeys reduced hepatic triglycerides and cholesterol, but Talh honey had superior effects on liver enzymes (ALT, AST, γ-GTT), inflammatory cytokines (TNF-α, IL-6), and oxidative stress markers (MDA, GSH, SOD). Talh honey also enhanced hepatic nuclear Nrf2 levels and AMPK signaling in the liver and white adipose tissue. These findings indicate that Talh honey exhibits more potent anti-obesity, hepatoprotective, antioxidant, and anti-inflammatory effects than Sidr honey, likely via modulation of AMPK and Nrf2 pathways.

## 1 Introduction

Obesity is a major contributor to increased morbidity and mortality, representing a significant public health issue and a heightened risk of non-communicable diseases, including type 2 diabetes, hypertension, and certain cancers (1, 2). In Saudi Arabia, unhealthy Western lifestyles have led to a high obesity prevalence, estimated at 33.7% in 2016, with about 66%–75% of adults in the Arab region classified as overweight or obese (3). This ongoing crisis underscores the need for effective preventive measures.

Obesity is directly associated with excessive calorie intake and is a significant contributor to liver disease development (4). A high-fat diet (HFD) primarily fosters weight gain through enhanced adipogenesis and impaired lipolysis. Increased dietary lipid availability promotes adipogenesis, which is the process of transforming preadipocytes into mature adipocytes, thereby resulting in greater fat storage in adipose tissue. This process is regulated by essential transcription factors, including sterol regulatory element-binding protein 1 (SREBP-1), peroxisome proliferator-activated receptors alpha and gamma (PPARα and PPARγ), and CCAAT/enhancer-binding protein alpha (C/EBPα), which activate genes critical for fat cell development (5, 6). Concurrently, HFD inhibits lipolysis, the catabolism of stored fats, by enhancing the activity of acetyl-CoA carboxylase (ACC) and fatty acid synthase (FAS) and lowering the activity of hormone-sensitive lipase (HSL) (5, 7, 8). Consequently, the accumulation of white adipose tissue (WAT) can cause systemic inflammation and insulin resistance (IR) (9, 10), both of which are major risk factors for non-alcoholic fatty liver disease (NAFLD) and its severe form, non-alcoholic steatohepatitis (NASH). Excessive fat accumulation in the liver disrupts metabolic processes, leading to lipotoxicity, mitochondrial damage, hepatic IR, oxidative stress, and inflammation (11, 12). This condition is further exacerbated by the release of free fatty acids and pro-inflammatory cytokines from visceral fat, which promotes hepatic steatosis and NASH progression through activation of the NF-κB pathway and endoplasmic reticulum stress (11, 13).

In recent years, accumulating experimental and clinical evidence has highlighted the activation of AMP-activated protein kinase (AMPK) as a promising strategy for enhancing metabolic health, particularly in promoting weight loss and managing obesity-associated disorders such as insulin resistance (IR), non-alcoholic fatty liver disease (NAFLD), and non-alcoholic steatohepatitis (NASH). As a crucial energy sensor, elevated AMP/ATP ratios activate AMPK during energy shortages (14). Once activated, it suppresses lipogenesis while facilitating glucose transporter expression (GLUT-4) in adipose and muscle, increasing glucose uptake, and promoting fatty acid oxidation (14, 15). On the contrary, reduced AMPK activity in WAT is linked to systemic insulin resistance in obese individuals, highlighting its pivotal role in NAFLD development (16, 17). AMPK-deficient mice exhibit glucose intolerance and increased fat deposition alongside WAT hypertrophy (18). Moreover, animal models with impaired liver-specific AMPK activity reveal elevated lipid accumulation, steatosis, and inflammation (19). Several studies confirmed that activated AMPK has a protective role in combating weight gain and NAFLD progression (20, 21). Overall, the mechanisms through which AMPK confers benefits include inhibition of lipogenesis and promotion of mitochondrial health and fatty acid oxidation (22, 23). Additionally, AMPK mitigates fat accumulation and insulin resistance by downregulating lipogenesis and adipogenesis while enhancing lipolysis (22, 23). Its regulatory effects extend to several critical targets within WAT and hepatic tissues, including SREBP1, acetyl-CoA carboxylase, SREBP1, carnitine palmitoyltransferase I (CPT I), perilipin 1 (PLIN1), SIRT1, HSL (22–26).

Honey, a natural product produced by honeybees, is widely recognized for its nutritional benefits and contains over 200 bioactive compounds (27). Composed primarily of carbohydrates, specifically fructose and glucose, honey also includes proteins, amino acids, water, lipid-soluble vitamins, enzymes, trace elements, organic acids, alkaloids, and a variety of phenolic compounds such as flavonoids and phenolic acids (28). The composition and physical characteristics of honey can vary significantly based on geographic location, bee species, and the types of floral nectar collected (28). Additionally, types of phenolic compounds influence the color of honey (28). Globally recognized for its medicinal properties, honey facilitates wound healing and offers protection against respiratory, cardiovascular, gastrointestinal, and neurological conditions (29–35). Various studies indicate that natural and processed honey, such as Gelam and Tualang from Malaysia, Manuka from New Zealand, and other honey types (e.g., Brazilian, German, and Chinese) exhibit anti-obesity effects in both humans and animals through mechanisms like anti-adiposity and hypoglycemic actions (28, 36, 37). These therapeutic effects are largely attributed to the potent antioxidant and anti-inflammatory properties of honey’s phenolic compounds, which might act by several mechanisms such as scavenging reactive oxygen species and regulating various signaling pathways such as SREBP1/FAS, AMPK, the nuclear factor kappa beta (NF-κB), and the nuclear factor erythroid factor-2 (Nrf2)/antioxidant pathway (38–40).

In Saudi Arabia, the most prevalent and indigenous honeybee species is Apis mellifera jemenitica. Saudi honey has been classified based on its botanical sources and the season of nectar collection (41). Common seasonal honey varieties include Sidr, Talha, Sumra, Seyfi, and Majra, which are derived from plants such as Ziziphus spp., Acacia gerrardii, Acacia tortilis, Lavandula spp., and Hypoestes forskalii (41, 42). Notably, certain types of Saudi honey, particularly Sidr and Sumra, exhibit a wide range of medicinal properties, including antimicrobial, antioxidant, and anti-inflammatory effects. They also alleviate hepatic and nephrotoxicity and promote wound healing in diabetic patients (40–46).

However, the anti-obesity and metabolic effects of Saudi honey types on obesity-related dysfunctions remain under-explored and warrant further investigation. In this study, we tested and compared the Talh and Sidr, two Saudi honey common types against anti-obesity and hepatic steatosis in rats fed a high-fat diet (HFD). In addition, we examined their mechanisms of action by targeting their effects on AMPK and Nrf2/antioxidant signaling pathways.

## 2 Materials and methods

### 2.1 Animals

This study employed male Wistar albino rats (100 ± 15 g), obtained from the animal facility at King Saud University, Riyadh, Saudi Arabia. The animals were housed in groups of three to four per standard polycarbonate cage, using autoclaved wood shavings as bedding. Group housing was chosen to promote social interaction and reduce stress, in alignment with recommended animal welfare practices. The housing environment was maintained under controlled conditions (temperature: 20–22°C; humidity: 40%–60%) with a 12 h light/dark cycle to preserve circadian rhythm and physiological homeostasis. Environmental enrichment (e.g., paper rolls, nesting material) was provided to support natural behaviors and enhance animal wellbeing. All animals had unrestricted access to standard laboratory chow and fresh water throughout the study. After a 1 week acclimatization period, rats were randomly allocated into eight experimental groups (*n* = 8 per group) using a computer-generated random number table. Investigators conducting biochemical, molecular, and histological analyses were blinded to the treatment groups to minimize observer bias. All experimental procedures adhered to institutional and international guidelines for the ethical use of laboratory animals and were approved by the Research Ethics Committee at King Saud University, Riyadh, Saudi Arabia (Ethics reference No. KSU-SE-24-28).

### 2.2 Bee honey sample collection

Two different types of local bee honey, namely Talh (Acacia Gerardii) and Sidr (Ziziphus sp.), were freshly purchased from certified suppliers located in the southern parts of Saudi Arabia. The suppliers have received certification from the Ministry of Environment, Water, and Agriculture for each province. The Sidr and Talh honey were collected between September and April 2023 and stored at 4°C. The samples underwent additional component analysis at accredited laboratories in Riyadh, KSA. Honey samples were stored at 4°C in sealed, dark glass containers to protect them from light and air. Prior to administration, samples were inspected for crystallization and gently liquefied in a water bath (below 40°C) when necessary to preserve bioactivity and ensure homogeneity, following standard handling practices.”

### 2.3 Control and HFD composition

A high-fat diet (HFD) [Item No. D12492; Research Diets Inc. (New Brunswick, NJ, United States)], containing 35% fat with a caloric density of 5.24 kcal/g, was administered to induce obesity in Wistar rats. The HFD formulation included 245 g/kg of lard and 25 g/kg of soybean oil, providing a substantial increase in fat content compared to the standard diet (STD) (Item No. D12450K), which contained only 4.3% fat and a caloric density of 3.85 kcal/g. In addition to the elevated fat content, the HFD incorporated 172.8 g/kg of sucrose, further boosting its caloric value, while the standard diet did not contain sucrose. Both diets were carefully balanced with essential nutrients like casein, corn starch, maltodextrin, and a full complement of vitamins and minerals. Prior to dietary intervention, rats were acclimatized for 1 week to reduce stress and ensure a stable baseline before initiating the obesity-inducing regimen. Our laboratory has reliably used this HFD protocol to establish obesity in this rat strain over a period of 12–16 weeks, allowing for the assessment of adipogenesis and metabolic changes, as previously reported (47, 48).

### 2.4 Experimental groups and design

The animals were randomly assigned into eight experimental groups (*n* = 8 per group). The control group received a standard chow diet and 0.5 mL of normal saline orally every 2 days for 12 weeks. The HFD group was fed a high-fat diet (HFD) and concurrently administered 0.5 mL of normal saline on the same schedule. The remaining six groups received HFD along with oral administration of either Sidr or Talh honey at different doses. Specifically, three groups received Sidr honey at 500, 700, or 1,000 mg/kg, while the other three received Talh honey at the same respective doses. Honey was administered orally every 2 days using a stainless-steel cannula. Daily oral treatment of various types of honey, such as Nigerian, Tualang, and Trihoney honey, attenuated hepatic steatosis in HFD rats at doses of 400–1,200 mg/kg without any signs of toxicity (49, 50). In addition, treating HFD rats with Nigerian honey at a dose of 1,000 mg/kg attenuated weight gain and hyperlipidemia and improved serum total antioxidant levels in diabetic and obese rats (51, 52). Also, HFD rats given stingless bee honey at doses of 500, 750, and 1,000 mg/kg showed a dose-response improvement in obesity-related parameters (53). The appropriate dose of honey for each rat was calculated based on its individual body weight using the formula: Dose (mg/kg) × Body Weight (kg). The weighed amount of honey (e.g., 100 mg for a 200 g rat at 500 mg/kg) was diluted in 0.5 mL of normal saline for oral gavage. A uniform administration volume of 0.5 mL was used for all animals across treatment groups to ensure consistent delivery and handling.

### 2.5 Assessment of nutritional intake and body metrics in the rat model

Nutritional intake and body measurements were systematically monitored at 4 weeks intervals throughout the study using well-established and validated protocols (54, 55). The total energy consumption was estimated by multiplying the average daily food intake by the metabolizable energy of the diet. To assess body composition, body mass index (BMI) was calculated by dividing the animal’s body weight (g) by the square of its body length (cm^2^). The Lee index, a key indicator of obesity, was calculated by taking the cube root of body weight (g), dividing it by body length (cm), and multiplying by 1,000. Values exceeding 310% are considered indicative of obesity. All measurements were performed on eight rats per group.

### 2.6 Blood, liver, and WAT collection

Following the 12 weeks treatment period, the rats were subjected to an overnight fasting period. They were then anesthetized with a mixture of ketamine (80 mg/kg) and xylazine (10 mg/kg). Blood samples were obtained via cardiac puncture into non-additive and EDTA-additive tubes to collect serum and plasma, respectively. After allowing the samples to clot for 30 min at room temperature, they were then centrifuged (1,200 × *g*; 10 min) to separate the serum and plasma. The serum and plasma were stored at −20°C for future analysis. All euthanasia procedures, including neck dislocation, were conducted in strict compliance with ethical standards. In addition, liver tissue and multiple adipose depots, specifically subcutaneous (inguinal), epididymal, peritoneal, and mesenteric fat pads, were carefully weighed and cut into smaller sections. The sections were rapidly frozen at −80°C and kept for subsequent investigations. Fresh liver and WAT (mesenteric) samples were placed in neutral formalin and processed for histological evaluation.

### 2.7 Lipid extraction and liver homogenate preparation

Liver lipids were carefully extracted using the protocol of Folch et al. (56). Briefly, 250 mg of fresh liver tissue was homogenized for three cycles of 3 min each on ice using 5 mL of a chloroform/methanol (2:1, v/v) solution. The homogenate was then incubated at 4°C for 1 h, followed by centrifugation at 1,400 × *g* for 10 min. The lower organic phase, which contained lipids, was carefully removed and washed with an additional portion of solvent. The organic solvent evaporated under reduced pressure using a rotary evaporator. The dried lipid extract was then reconstituted in 200 μL of isopropanol for further analysis. On the other hand, other parts of livers (50 mg) were homogenized in 450 μL phosphate-buffered saline (PBS) at a high speed of 10,000 rpm for 30 s in the presence of 5 μL protease/phosphatase inhibitor cocktail (Item No. 5872, Cell Signaling Technology, Danvers, MA, United States) to prepare total cell homogenate for biochemical analyses. The resultant homogenates were centrifuged at a speed of 11,000 × *g* for 20 min to collect the supernatant, which was then stored for further measurement.

### 2.8 Biochemical analysis in plasma and serum

Plasma glucose levels were measured using a colorimetric assay (Item No. 10009582, Cayman Chemical, Ann Arbor, MI, United States). Insulin levels in the plasma samples were quantified using an ELISA kit (Item No. ERINS, Thermo Fisher, Bremen, Germany). Hemoglobin A1c (HbA1c) in plasma was assessed using an assay kit (Item No. 80300, Crystal Chem, IL, United States). Total free fatty acids (FFAs), cholesterol (CHOL), and triglycerides (TGs) in serum and liver homogenates were determined using assays (Item Nos. 700310, 10007640, 10010303, Cayman Chemical, Ann Arbor, MI, United States). Low-density lipoprotein cholesterol (LDL-c) and high-density lipoprotein cholesterol (HDL-c) levels in serum and hepatic homogenates were measured by ELISA kits specific for rats (Item No. E-EL-R0579, Elabscience, Houston, TX, United States; Item No. MBS266554, MyBiosource, San Diego, CA, United States). Serum levels of rat alanine aminotransferase (ALT) and aspartate aminotransferase (AST) were determined using rat-specific sandwich-based ELISA kits (Item Nos. ab285264 and ab263883, Abcam, Cambridge, United Kingdom). Serum levels of gamma glutamyl transferase (GTT) were measured an ELISA kit provided form MyBiosource, (San Diego, CA, United States) (Item No. MBS2881417). All kits were rat’s specific and all analysis were done in duplicate for eight samples per group.

### 2.9 Extraction of total proteins from WAT and liver and biochemical analysis

Total proteins were meticulously extracted from 2 g of mesenteric WAT or liver utilizing a specialized commercial protein extraction kit (Catalog ID AT-022, Invent Biotechnologies, Plymouth, MN, United States, and Catalog ID, BioRad, Ann Arbor, MI, United States, respectively), following the manufacturer’s instructions. ELISA kits were used to measure total liver protein extract, including tumor necrosis factor-alpha (TNF-α) and interleukin-6 (IL-6) (Item Nos. KRC3011 and Item No. ERA31RB, Thermo Fisher, Bremen, Germany). The levels of malondialdehyde (MDA), superoxide dismutase-1 (SOD1/Cu-Zn), glutathione peroxidase 1 (GPx1), catalase, Bcl2, Bax, and caspase-3 levels in total liver homogenates were quantified using ELISA kits (item Nos. ER1878, ER0332, ER0274, ER0264, ER0762, ER0512, and ER2018, FineTest, Wuhan, China). The levels of cytochrome-c in the cytoplasmic fraction were measured using an ELISA kit for rats (Item Nos. ER0893, FineTest, Wuhan, China). The levels of phosphorylated AMPKα1 and p-ACC were assessed in total protein extract with specific rat ELISA kits (Item NOs. LS-F36060; LS Bio, MA, USA) and (abx255889, Abbexa, IL, United States). All used kits were rat-specific, and all analyses were performed in duplicates of *n* = 8 samples/group as per recommended by the manufacturers.

### 2.10 Extraction of hepatic nuclear proteins and biochemical measurements

The nuclear extract was obtained from frozen liver tissue using a commercially available kit (Item No. 40010, Active Motif, Tokyo, Japan) according to the manufacturer’s instructions. Frozen liver samples were first thawed and minced into small pieces. The pieces were then homogenized in 500 μL of ice-cold PBS that had 1 mM of sodium fluoride and sodium orthovanadate to maintain protein stability. After that, the homogenate was mixed with 500 μL of a hypotonic 10 mM HEPES (pH 7.9) lysis buffer containing 1.5 mM MgCl2, 10 mM KCl, and 0.5% NP-40. The mixture was put on ice for 15 min. Next, the sample was centrifuged at 1,000 × *g* for 5 min at 4°C to separate the cytoplasmic and nuclear fractions. The cytoplasmic fraction was carefully collected and stored at −80°C for later use. The nuclear pellet was resuspended in 100 μL of detergent-free lysis buffer. This buffer contained 20 mM HEPES (pH 7.9), 400 mM NaCl, 1.5 mM MgCl_2_, and a protease inhibitor cocktail. To yield the nuclear extract, the mixture was incubated on ice for 30 min, vortexed intermittently, and then centrifuged at 14,000 × *g* for 10 min at 4°C. Protein quantification was conducted calorimetrically employing the quick start Bradford protein Assay kit (Catalog ID 5000201EDU, BioRad, Feldkirchen, Germany). The levels of Nrf2 in the liver nuclear extracts were assessed using special ELISA kits provided by Active Motif, Carlsbad, CA, United States (Catalog IDs 50296 and 31102, respectively). All analyses were performed in duplicates of *n* = 8 samples per group following each kit manufacturer’s instructions.

### 2.11 Real-time polymerase chain reaction (qPCR)

To evaluate the mRNA expression of AMPK and other lipid-related genes, total RNA from mesenteric WAT and liver samples was isolated using the RNeasy Mini Kit (Catalog No. 74804, QIAGEN, Hilden, Germany), following the manufacturer’s protocol. RNA quality and concentration were assessed using a NanoDrop spectrophotometer (Thermo Fisher Scientific, Waltham, MA, United States). For cDNA synthesis, 1 μg of RNA was reverse transcribed using the High-Capacity cDNA Reverse Transcription Kit (Catalog No. 20531, Thermo Fisher Scientific, Foster City, CA, United States) in a total reaction volume of 20 μL, incubated at 37°C for 60 min, and subsequently at 95°C for 5 min. Quantitative real-time PCR (qPCR) was performed using the CFX96 Touch Real-Time PCR Detection System (Bio-Rad Laboratories, Hercules, CA, United States). The 20 μL PCR reaction contained 10 μL of 2x SYBR Green PCR Master Mix [Applied Biosystems (Foster City, CA, United States)], 0.5 μM of each primer, and 2 μL of cDNA. The thermal cycling conditions included an initial denaturation step at 95°C for 10 min, followed by 40 cycles of 95°C for 15 s and 60°C for 1 min. Relative gene expression was calculated using the ΔΔCt method, with β-actin as the reference gene. The primers used for gene amplification included AMPKα2 (NM_023991.1) with forward primer GTGGATCGCCAAATTATGCA and reverse primer AACCTCAGGACCCGCATACA (product size: 65 bp) and Nrf2 (NM_031789) with forward primer AAAATCATTAACCTCCCTGTTGAT and reverse primer CGGCGACTTTATTCTTACCTCT (product size: 118 bp). β-actin (NM_031144.3) was used as the reference gene with forward primer AGGCCCCTCTGAACCCTAAG and reverse primer CAGCCTGGATGGCTACGTACA (product size: 96 bp). Reactions were performed in duplicate for each group (*n* = 6) to ensure robust data consistency.

### 2.12 Histological study

Liver tissue samples were fixed in formalin, dehydrated through a graded ethanol series (70%, 80%, 90%, 95%, and 100%), and then embedded in paraffin. Sections (5–7 μm) were cut, mounted on slides, deparaffinized with xylene, and then rehydrated in descending ethanol concentrations (100%, 95%, 90%, 80%, and 70%). The liver sections were stained with hematoxylin for 5–10 min. The excess staining was then removed with an HCl-alcohol solution. Subsequently, stained liver samples were rinsed in distilled water. Eosin solution was applied for 1–3 min to stain the cytoplasm and extracellular matrix. Slides were dehydrated through ascending ethanol grades, cleared in xylene, and mounted with coverslips. The slides were then examined under a light microscope for histological analysis.

### 2.13 Statistical analysis

All collected data underwent statistical analysis using GraphPad Prism (Version 8). Normality was assessed with the Kolmogorov-Smirnov test. Analysis was conducted using one-way ANOVA, and significance levels were determined using Tukey’s post hoc test (*p* < 0.05). Results are presented as means ± standard deviation (SD) for all data sets.

## 3 Results

### 3.1 The honey attenuates HFD-induced gain in body and fat weights

Feeding rats a high-fat diet (HFD) led to a marked increase in body weight, total weight gain, food intake, body mass index (BMI), Lee index, and total fat mass relative to the control group. Significant hypertrophy of adipose tissue was observed in all measured fat depots, including mesenteric, subcutaneous, peritoneal, and epididymal fat, thereby confirming the obesogenic impact of HFD feeding (Table 1). Sidr honey supplementation at all tested doses (500, 700, and 1,000 mg/kg) did not significantly alter any of the obesity-related parameters compared to the HFD group. Notably, Sidr honey failed to mitigate weight gain or reduce adiposity despite its high sugar content. Interestingly, Talh honey exerted a dose-dependent anti-obesity effect. At 500 mg/kg, Talh honey significantly decreased body weight, weight gain, BMI, and several fat depot masses, including mesenteric, subcutaneous, and epididymal fat. These effects were further amplified at the 700 mg/kg dose, with substantial reductions in both body weight and adipose tissue accumulation. The most pronounced anti-obesity effects were observed at the 1,000 mg/kg dose, which elicited significant improvements across all measured parameters, including body weight, BMI, Lee index, and regional fat mass. Importantly, food intake did not differ significantly between HFD-fed rats and those treated with either Sidr or Talh honey, indicating that the observed reductions in body weight were independent of energy intake. No significant changes were detected between the HFD + Talh honey 100 mg/kg group and control rats across measured parameters (Table 1).

**TABLE 1:** Effect of Sidr and Talh honey on body weight, food intake, and fat distribution in high-fat diet (HFD)-induced obese rats.

Parameter	Control	HFD	HFD + Sidr (500 mg/kg)	HFD + Sidr (700 mg/kg)	HFD + Sidr (1,000 mg/kg)	HFD + Talh (500 mg/kg)	HFD + Talh (700 mg/kg)	HFD + Talh (1,000 mg/kg)
Initial body weights (g)	118.4 ± 13.2	110.5 ± 9.4	121.2 ± 11.4	116.4 ± 10.4	119.9 ± 11.7	122.2 ± 13.4	115.7 ± 11.6	120.4 ± 14.3
Final body weights (g)	448.4 ± 41	627.4 ± 51^a^	666.3 ± 57^a^	634.4 ± 61^a^	643.3 ± 33.6^a^	564.3 ± 41^abcde^	493 ± 33.6^abcdef^	418.4 ± 35^bcdefg^
Weight gain (%)	271.4 ± 19	472.4 ± 45^a^	453.2 ± 51^a^	432.4 ± 39^a^	439.8 ± 44^a^	366.4 ± 31^abcde^	318.3 ± 27^abcdef^	278.4 ± 21^bcdefg^
Average food intake (kg/last month/group)	7.8 ± 0.8	11.8 ± 1.6^a^	10.9 ± 1.2^a^	12.6 ± 1.7^a^	11.4 ± 1.2^a^	10.9 ± 1.4^a^	12.5 ± 1.7^a^	11.5 ± 1.4^a^
BMI (g/cm^2^)	0.61 ± 0.06	0.88 ± 0.08^a^	0.81 ± 0.09^a^	0.91 ± 0.1^a^	0.85 ± 0.09^a^	0.78 ± 0.08^abcde^	0.69 ± 0.05^abde^	0.58 ± 0.04^bcdefg^
Lee index	223.5 ± 21	364.3 ± 19^a^	359.1 ± 37^a^	355.5 ± 31^a^	366.3 ± 29^a^	301 ± 27^abcde^	265 ± 24^abcdef^	218 ± 24^abcdefg^
Mesenteric fat (g)	6.3 ± 0.56	13.6 ± 1.2^a^	12.6 ± 1.4^a^	13.8 ± 1.3^a^	12.4 ± 1.4^a^	10.9 ± 0.89^abcde^	8.1 ± 0.73^bcdef^	6.1 ± 0.54^abcdefg^
Subcutaneous fat (g)	7.4 ± 0.95	16.5 ± 0^a^	17.5 ± 1.7^a^	15.4 ± 1.5^a^	16.1 ± 1.8^a^	13.1 ± 1.1^abcde^	10.4 ± 0.94^bcdef^	7.1 ± 0.54^abcdefg^
Peritoneal fat (g)	6.4 ± 0.89	12.8 ± 1.6^a^	11.8 ± 1.3^a^	12.7 ± 1.5^a^	11.6 ± 1.5^a^	10.3 ± 1.1^abcde^	8.4 ± 0.71^bcdef^	6.1 ± 0.68^abcdefg^
Epididymal fat (g)	5.7 ± 0.62	9.2 ± 0.43^a^	9.3 ± 0.82^a^	9.7 ± 0.88^a^	10.1 ± 1.3^a^	8.1 ± 0.74^abcde^	7.3 ± 0.63^bcdef^	5.4 ± 0.54^abcdefg^
Total fat weight (g)	25.6 ± 2.5	49.3 ± 4.1^a^	50.9 ± 5.7^a^	48.1 ± 4.8^a^	52.4 ± 4.1^a^	40.9 ± 3.1^abcde^	33.2 ± 2.7^bcdef^	24.5 ± 2.6^abcdefg^

Data are presented as mean ± standard deviation (*n* = 8). Significant differences from the control group are indicated by superscript letters: a (vs. control), b (vs. HFD), c (vs. HFD + Sidr 500 mg/kg), d (vs. HFD + Sidr 700 mg/kg), e (vs. HFD + Sidr 1,000 mg/kg), f (vs. HFD + Talh 500 mg/kg), g (vs. HFD + Talh 700 mg/kg).

### 3.2 Honey influences plasma Glucose, serum Lipids, and adiponectin

Compared to the control group, HFD feeding resulted in significant elevations in key metabolic parameters, including fasting glucose, fasting insulin, HOMA-IR index, serum HbA1c, leptin, triglycerides, total cholesterol, and LDL-c levels. In contrast, serum adiponectin levels were markedly reduced, indicating the onset of insulin resistance and dyslipidemia (Table 2). Administration of Sidr honey to HFD-fed rats at doses of 500, 700, and 1,000 mg/kg significantly lowered plasma fasting glucose, HbA1c, and HOMA-IR in a dose-dependent manner. However, no significant changes were observed in fasting insulin, leptin, or adiponectin levels in Sidr-treated groups compared to HFD controls. In addition, Sidr honey effectively reduced serum triglyceride, cholesterol, and LDL-c concentrations in a dose-dependent fashion, reflecting its beneficial effects on lipid homeostasis (Table 2). Talh honey demonstrated more robust metabolic improvements across all doses tested. Treatment with Talh honey significantly decreased fasting plasma glucose, insulin, HOMA-IR, HbA1c, leptin, triglycerides, cholesterol, and LDL-c levels in a dose-dependent manner. Furthermore, Talh honey significantly increased serum adiponectin levels in HFD-fed rats, indicating enhanced insulin sensitivity and anti-inflammatory potential (Table 2). HFD feeding also resulted in significant hepatic accumulation of cholesterol and triglycerides. Both Sidr and Talh honey significantly reduced hepatic lipid accumulation, with Talh honey (especially at 1,000 mg/kg) restoring hepatic cholesterol and triglyceride levels to near-control values (Table 2).

**TABLE 2:** Impact of Sidr and Talh honey on metabolic parameters in high-fat diet (HFD)-induced obesity in rats.

Parameter	Control	HFD	HFD + Sidr (500 mg/kg)	HFD + Sidr (700 mg/kg)	HFD + Sidr (1,000 mg/kg)	HFD + Talh (500 mg/kg)	HFD + Talh (700 mg/kg)	HFD + Talh (1,000 mg/kg)
Fasting glucose (mg/dL)	92.2 ± 8.5	223.4 ± 14.5^a^	189.3 ± 17.9^ab^	152.3 ± 16.5^abc^	122.4 ± 13.5^abcd^	154.5 ± 12.6^abcde^	126.2 ± 15.8^abcdef^	101.7 ± 105 ^bcdefg^
Fasting insulin (ng/mL)	3.6 ± 0.49	8.5 ± 0.91	8.1 ± 0.95^a^	8.9 ± 0.81^a^	8.4 ± 0.87^a^	6.7 ± 0.42^abcde^	5.1 ± 0.64^abcdef^	3.3 ± 0.54^bcdefg^
HOMA-IR	0.83 ± 0.09	4.7 ± 0.6^a^	3.6 ± 0.37^ab^	3.4 ± 0.54^ab^	2.41 ± 0.39^abcd^	2.5 ± 0.31^abcd^	1.52 ± 0.26^abcdef^	0.85 ± 0.6^bcdefg^
Serum HbA1C (%)	3.78 ± 0.54	8.5 ± 0.79^a^	7.41 ± 0.63^ab^	6.2 ± 0.53^abc^	5.1 ± 0.62^abcd^	6.8 ± 0.73^abde^	5.1 ± 0.45^abcdef^	3.9 ± 38^bcdefg^
Serum leptin (ng/mL)	56.2 ± 5.8	99.4 ± 10.1^a^	101.5 ± 10.9^a^	95.6 ± 9.1^a^	106.2 ± 9.4^a^	86.5 ± 7.5^abcde^	71.3 ± 6.1^abcdef^	52.3 ± 6.8^bcdefg^
Serum adiponectin (μg/mL)	108.1 ± 11.2	45.2 ± 7.5^a^	47.3 ± 5.8^a^	44.7 ± 5.6^a^	49.3 ± 5.3^a^	75.6 ± 5.4^abcde^	88.4 ± 7.3^abcdef^	112.4 ± 11.4^bcdefg^
Serum Triglycerides (mg/dL)	93.2 ± 8.1	254.1 ± 21.4^a^	228.2 ± 23.2^ab^	198.3 ± 17.6^abc^	165.2 ± 15.4^abcd^	202.1 ± 18.5^abde^	135.4 ± 0.73^bcdef^	97.6 ± 8.2^bcdefg^
Serum cholesterol (mg/kg)	77.3 ± 5.7	202.2 ± 19.3^a^	178.5 ± 14.8^ab^	152.3 ± 15.7^abc^	143.2 ± 12.3^abcd^	145.4 ± 10.5^abde^	104.3 ± 11.3^bcdef^	75.6 ± 6.8^bcdefg^
Serum LDL-c (mg/dL)	33.6 ± 4.5	105.2 ± 8.2^a^	86.6 ± 8.4^ab^	76.4 ± 6.4^abc^	63.4 ± 5.6^abcd^	75.6 ± 6.4^abde^	51.6 ± 5.4^bcdef^	37.3 ± 3.6^bcdefg^
Hepatic triglycerides (mg/g liver)	3.3 ± 0.43	9.3 ± 0.82^a^	7.6 ± 0.54^ab^	6.1 ± 0.74^bc^	5.1 ± 0.48^abcd^	6.11 ± 0.58^abde^	4.33 ± 0.44^bcdef^	3.2 ± 0.41^bcdefg^
Hepatic cholesterol (μg/g liver)	1.8 ± 0.22	5.9 ± 0.44^a^	4.8 ± 0.32^ab^	3.6 ± 0.36^bc^	2.5 ± 0.44^abcd^	4.1 ± 0.39^abde^	2.6 ± 0.39^bcdef^	1.77 ± 0.11^bcdefg^

Data are presented as mean ± standard deviation (*n* = 8). Significant differences from the control group are indicated by superscript letters: a (vs. control), b (vs. HFD), c (vs. HFD + Sidr 500 mg/kg), d (vs. HFD + Sidr 700 mg/kg), e (vs. HFD + Sidr 100 mg/kg), f (vs. HFD + Talh 500 mg/kg), g (vs. HFD + Talh 700 mg/kg).

### 3.3 The honey effect on hepatic lipids

High-fat diet feeding caused a marked elevation in hepatic cholesterol and triglyceride levels compared to the control group, confirming the development of hepatic steatosis (Table 2). Treatment with Sidr honey at doses of 500, 700, and 1,000 mg/kg significantly reduced hepatic cholesterol and triglyceride levels in a dose-dependent manner. However, Talh honey exhibited a more pronounced hepatoprotective effect. Across all tested doses, Talh honey led to greater reductions in hepatic lipid accumulation, with the 1,000 mg/kg dose restoring both hepatic cholesterol and triglyceride levels to values comparable to those observed in the control group. These findings suggest that Talh honey is more effective than Sidr honey in ameliorating hepatic lipid dysregulation induced by high-fat diet feeding (Table 2).

### 3.4 Honey mitigates liver dysfunction and serum inflammatory markers

High-fat diet-fed rats exhibited a significant increase in serum levels of liver enzymes (ALT, AST, and γ-GTT) and pro-inflammatory cytokines (TNF-α and IL-6) compared to the control group, indicating hepatic injury and systemic inflammation (Table 3). Administration of Sidr honey at 500, 700, and 1,000 mg/kg to HFD-fed rats resulted in dose-dependent reductions in ALT, AST, and γ-GTT. However, Sidr honey exhibited a limited effect on inflammatory cytokines at lower doses; only the 1,000 mg/kg dose significantly reduced TNF-α and IL-6 levels. In contrast, Talh honey produced more pronounced hepatoprotective and anti-inflammatory effects across all doses. Treatment with Talh honey markedly improved liver enzyme profiles and significantly suppressed TNF-α and IL-6 levels, with the highest dose (1,000 mg/kg) restoring most parameters to levels comparable to those of control rats. These results demonstrate that while both types of honey confer hepatic protection, Talh honey exerts superior efficacy, particularly in mitigating inflammation and normalizing liver function markers in the context of HFD-induced liver injury.

**TABLE 3:** Effect of Sidr and Talh honey on serum levels of liver enzymes and inflammatory markers in high-fat diet (HFD)-induced obese rats.

Parameter	Control	HFD	HFD + Sidr (500 mg/kg)	HFD + Sidr (700 mg/kg)	HFD + Sidr (1,000 mg/kg)	HFD + Talh (500 mg/kg)	HFD + Talh (700 mg/kg)	HFD + Talh (1,000 mg/kg)
ALT (U/L)	18.3 ± 1.1	98.3 ± 8.3^a^	65.9 ± 6.1^ab^	42.2 ± 5.1^abc^	33.5 ± 4.7^abcd^	61.2 ± 7.4^abcde^	32.3 ± 2.5^bcdef^	16.7 ± 1.2^bcdefg^
AST (U/mL)	37.4 ± 2.5	145.4 ± 12.3^a^	122.2 ± 11.2^ab^	97.4 ± 10.1^abc^	82.3 ± 7.8^abcd^	102.1 ± 8.4^abcde^	63.4 ± 5.8^abcdef^	39.4 ± 5.3^bcdefg^
GTT (U/L)	22.3 ± 2.1	74.3 ± 6.5^a^	65.6 ± 4.5^ab^	57.5 ± 5.8^abc^	46.5 ± 4.3^abcd^	54.3 ± 4.1^abcd^	33.3 ± 3.2^abcdef^	24.3 ± 1.8^bcdefg^
TNF-α (pg/mL)	4.3 ± 0.74	38.5 ± 3.3^a^	34.4 ± 3.1^a^	30.2 ± 2.4^a^	28.4 ± 2.5^abc^	22.4 ± 1.2^abcde^	11.2 ± 1.3^abcdef^	5.3 ± 0.83^bcdefg^
IL-6 (pg/mL)	22.3 ± 2.8	134.5 ± 14.2^a^	127.3 ± 12.3^a^	122.3 ± 11.9^a^	102.3 ± 11.4^abcd^	87.5 ± 7.7^abcd^	34.3 ± 2.7^abcdef^	20.5 ± 2.4^bcdefg^

Data are presented as mean ± standard deviation (*n* = 8). Significant differences from the control group are indicated by superscript letters: a (vs. control), b (vs. HFD), c (vs. HFD + Sidr, 500 mg/kg), d (vs. HFD + Sidr 700 mg/kg), e (vs. HFD + Sidr, 1,000 mg/kg), f (vs. HFD + Talh, 500 mg/kg), g (vs. HFD + Talh, 700 mg/kg).

### 3.5 Antioxidant and anti-inflammatory effects of honey

**FIGURE 1:**
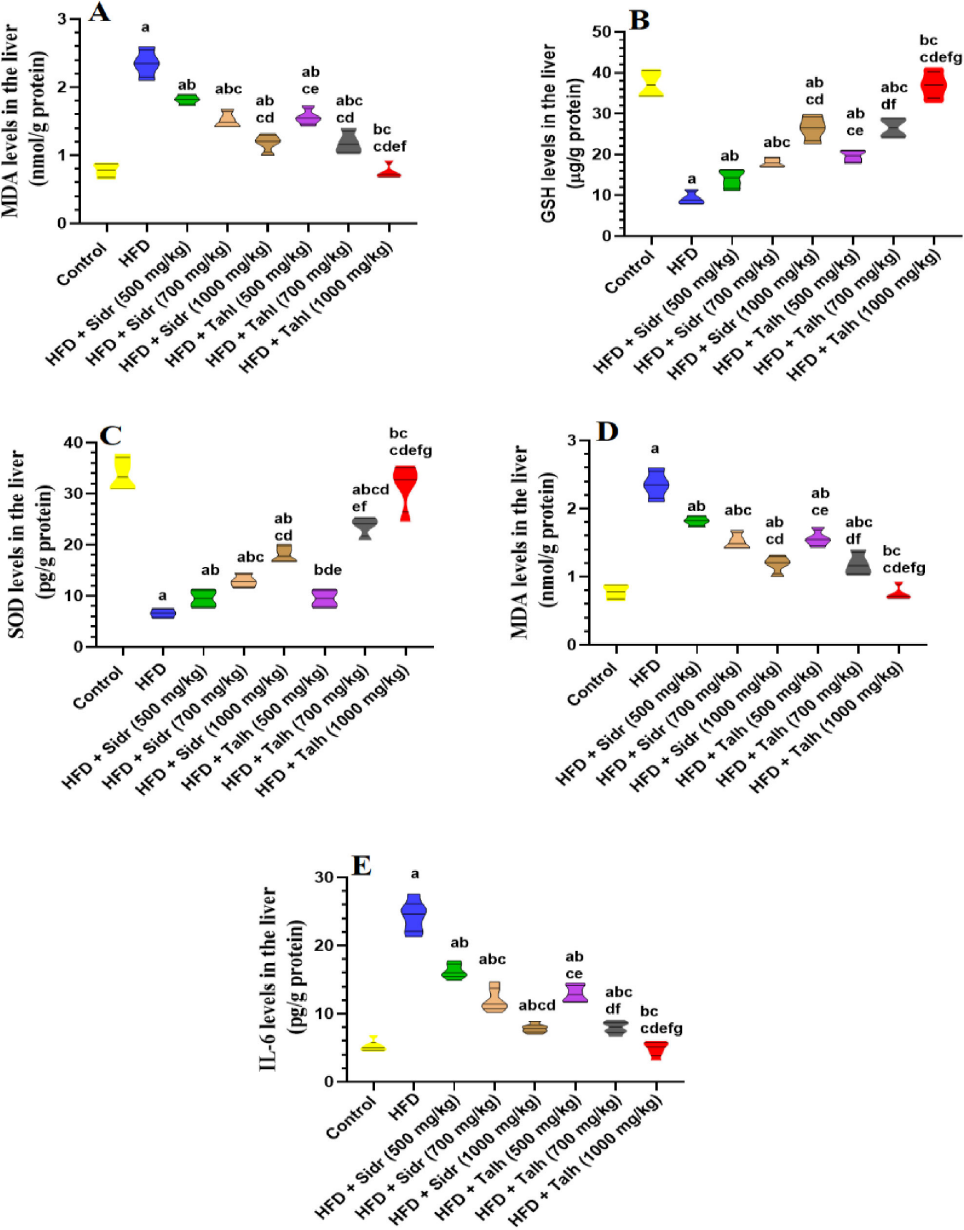
Impact of Sidr and Talh honey on oxidative stress **(A–D)** and inflammation markers **(E)** in the livers of all groups of rats. Data are presented as mean ± standard deviation (*n* = 8). Significant differences from the control group are indicated by superscript letters: a (vs. control), b (vs. HFD), c (vs. HFD + Sidr 500 mg/kg), d (vs. HFD + Sidr 700 mg/kg), e (vs. HFD + Sidr 1,000 mg/kg), f (vs. HFD + Talh 500 mg/kg), g (vs. HFD + Talh 700 mg/kg).

Livers of HFD-fed rats showed marked elevations in MDA, TNF-α, and IL-6, alongside significant reductions in GSH and SOD, indicating increased oxidative stress and inflammation (Figures 1A–E). Treatment with Sidr honey at 500 mg/kg moderately improved these markers by reducing MDA, TNF-α, and IL-6, while increasing GSH and SOD levels. These effects became more pronounced at 700, and 1,000 mg/kg.

In contrast, Talh honey exerted stronger and dose-dependent effects, significantly lowering MDA, TNF-α, and IL-6, and markedly enhancing GSH and SOD levels across all doses. At 1,000 mg/kg, Talh honey restored these parameters to levels comparable with control rats, suggesting a potent hepatoprotective effect.

### 3.6 Honey stimulates Nrf2 and alleviates apoptosis in the livers of HFD rats

**FIGURE 2:**
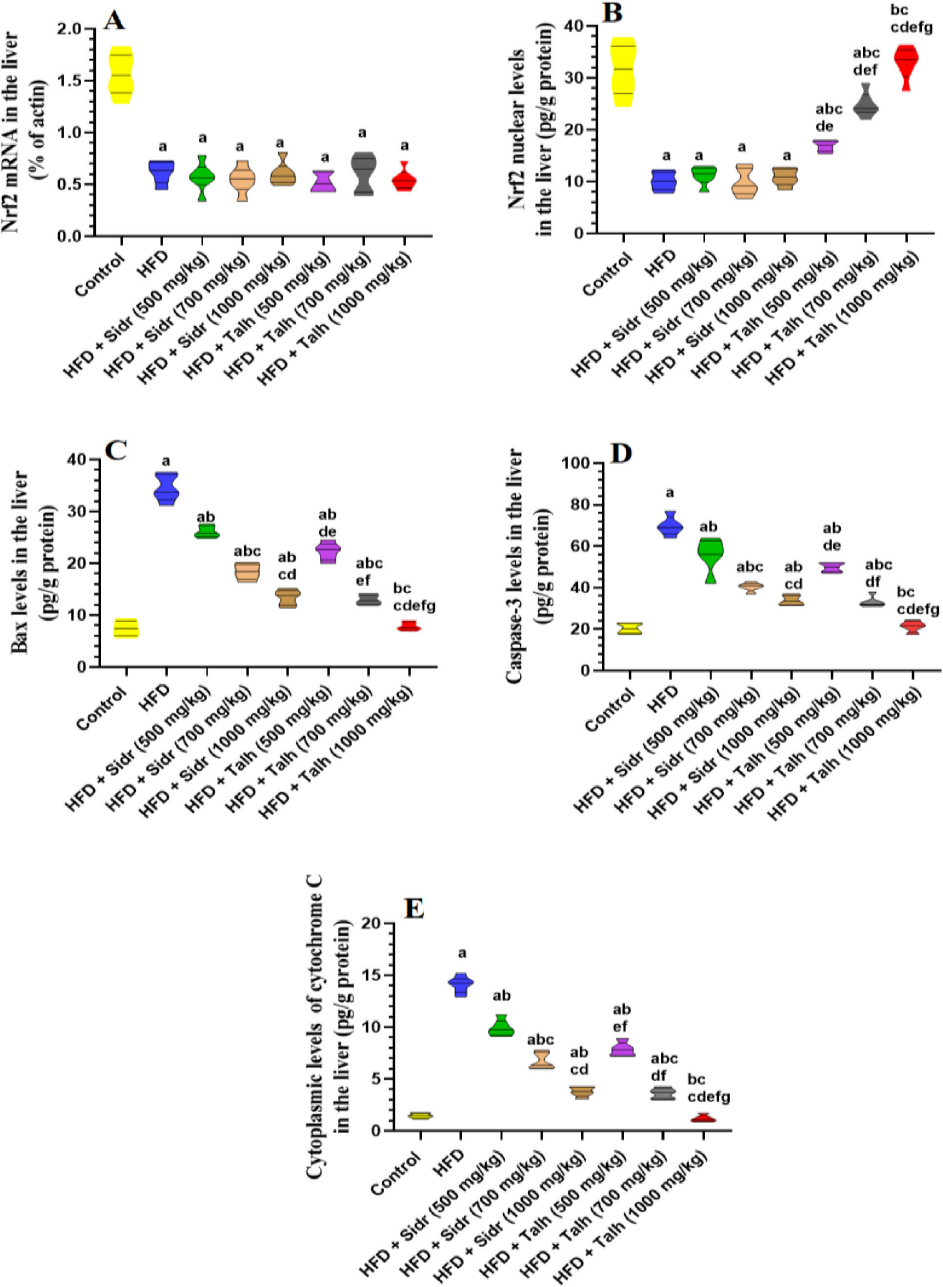
Impact of Sidr and Talh honey on expression and nuclear levels of Nrf2 **(A,B)** and on some markers of apoptosis **(C–E)** in the livers of all groups of rats. Data are presented as mean ± standard deviation (*n* = 8). Significant differences from the control group are indicated by superscript letters: a (vs. control), b (vs. HFD), c (vs. HFD + Sidr 500 mg/kg), d (vs. HFD + Sidr 700 mg/kg), e (vs. HFD + Sidr 1,000 mg/kg), f (vs. HFD + Talh 500 mg/kg), g (vs. HFD + Talh 700 mg/kg).

All treatments did not significantly alter hepatic mRNA levels of Nrf2 compared to control rats (Figure 2A). However, HFD markedly reduced nuclear Nrf2 protein levels relative to control (Figure 2B), which was accompanied by elevated hepatic levels of Bax, caspase-3, and cytosolic Cyt-c (Figures 2C–E), indicating oxidative stress and enhanced apoptosis. Sidr honey at all tested doses (500, 700, and 1,000 mg/kg) did not significantly restore nuclear Nrf2 levels relative to HFD (Figure 2B), but it exerted a dose-dependent improvement in apoptotic markers. Administration of Sidr honey led to progressive reductions in hepatic Bax, caspase-3, and Cyt-c levels in comparison to HFD rats (Figures 2C–E), suggesting attenuation of apoptosis. In contrast, Talh honey significantly increased nuclear Nrf2 protein levels in a dose-dependent manner. It also resulted in a marked reduction in hepatic Bax, caspase-3, and Cyt-c levels across all tested doses (Figures 2C–E). These findings indicate that Talh honey effectively enhanced Nrf2 signaling and suppressed apoptotic activity in the liver of HFD-fed rats.

### 3.7 Honey stimulates AMPK signaling in the liver and WAT of HFD rats

**FIGURE 3:**
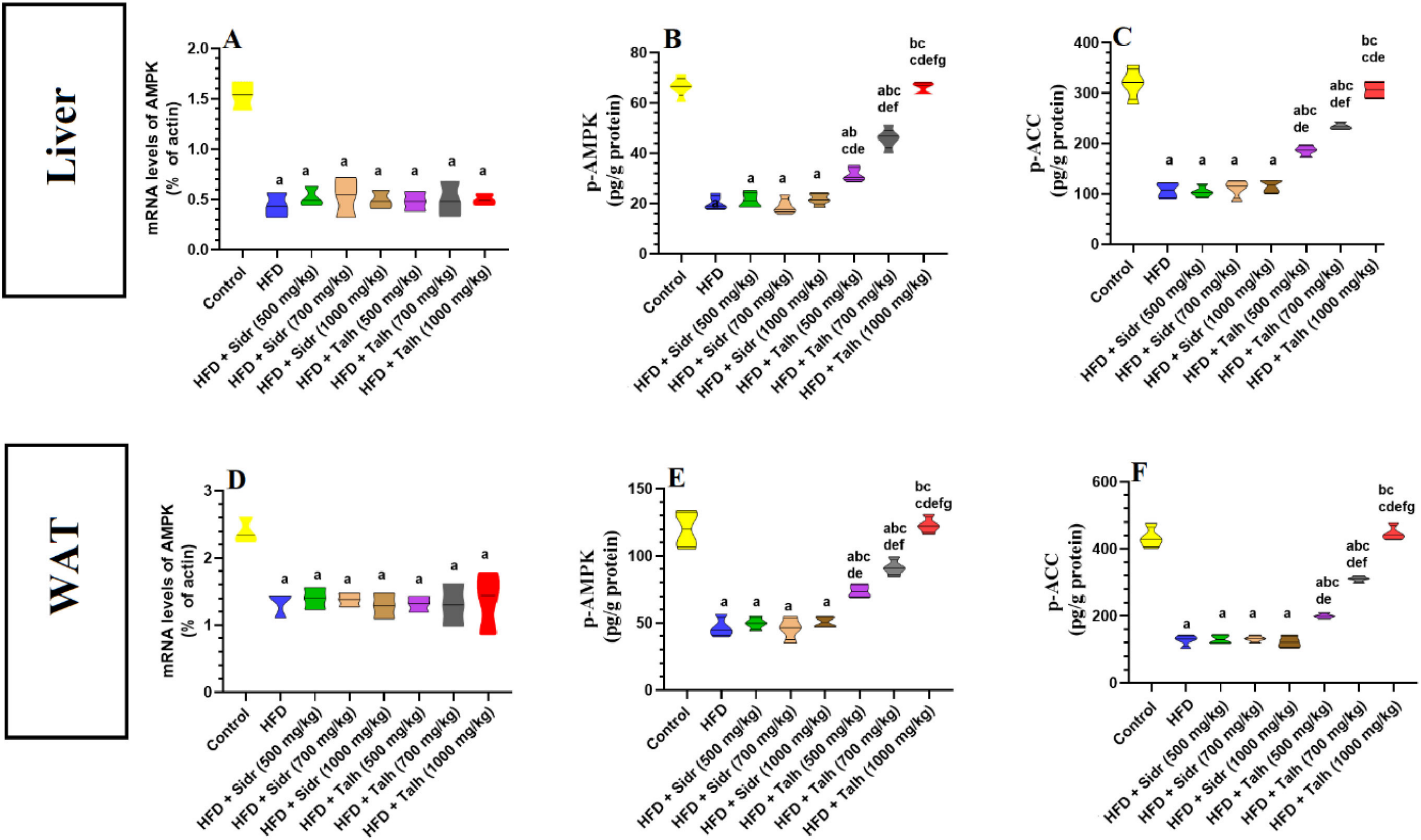
Impact of Sidr and Talh honey on the expression and activity of AMPK and on the phosphorylation levels of Acetyl CoA Carboxylase (ACC) **(A–F)** in the livers of all groups of rats. Data are presented as mean ± standard deviation (*n* = 8). Significant differences from the control group are indicated by superscript letters: a (vs. control), b (vs. HFD), c (vs. HFD + Sidr 500 mg/kg), d (vs. HFD + Sidr 700 mg/kg), e (vs. HFD + Sidr 1,000 mg/kg), f (vs. HFD + Talh 500 mg/kg), g (vs. HFD + Talh 700 mg/kg).

The HFD group exhibited markedly reduced hepatic mRNA levels of AMPK, p-AMPK, and p-ACC compared to the control group (Figures 3A–C). Administration of Sidr honey at doses of 500, 700, and 1,000 mg/kg did not significantly affect the hepatic levels of these markers relative to HFD. In contrast, treatment with Talh honey at all tested doses led to clear upregulation of hepatic AMPK, p-AMPK, and p-ACC expression in HFD-fed rats (Figures 3A–C).

Similarly, in white adipose tissue (WAT), the HFD group showed notable reductions in AMPK, p-AMPK, and p-ACC mRNA levels compared to controls (Figures 3D–F). Sidr honey treatment at all tested doses failed to restore these levels. On the other hand, Talh honey produced a progressive and dose-dependent elevation in the expression of AMPK pathway components in WAT, particularly at higher doses, indicating systemic activation of the AMPK/ACC axis (Figures 3D–F).

### 3.8 Honey impacts the histology of HFD rat livers

**FIGURE 4:**
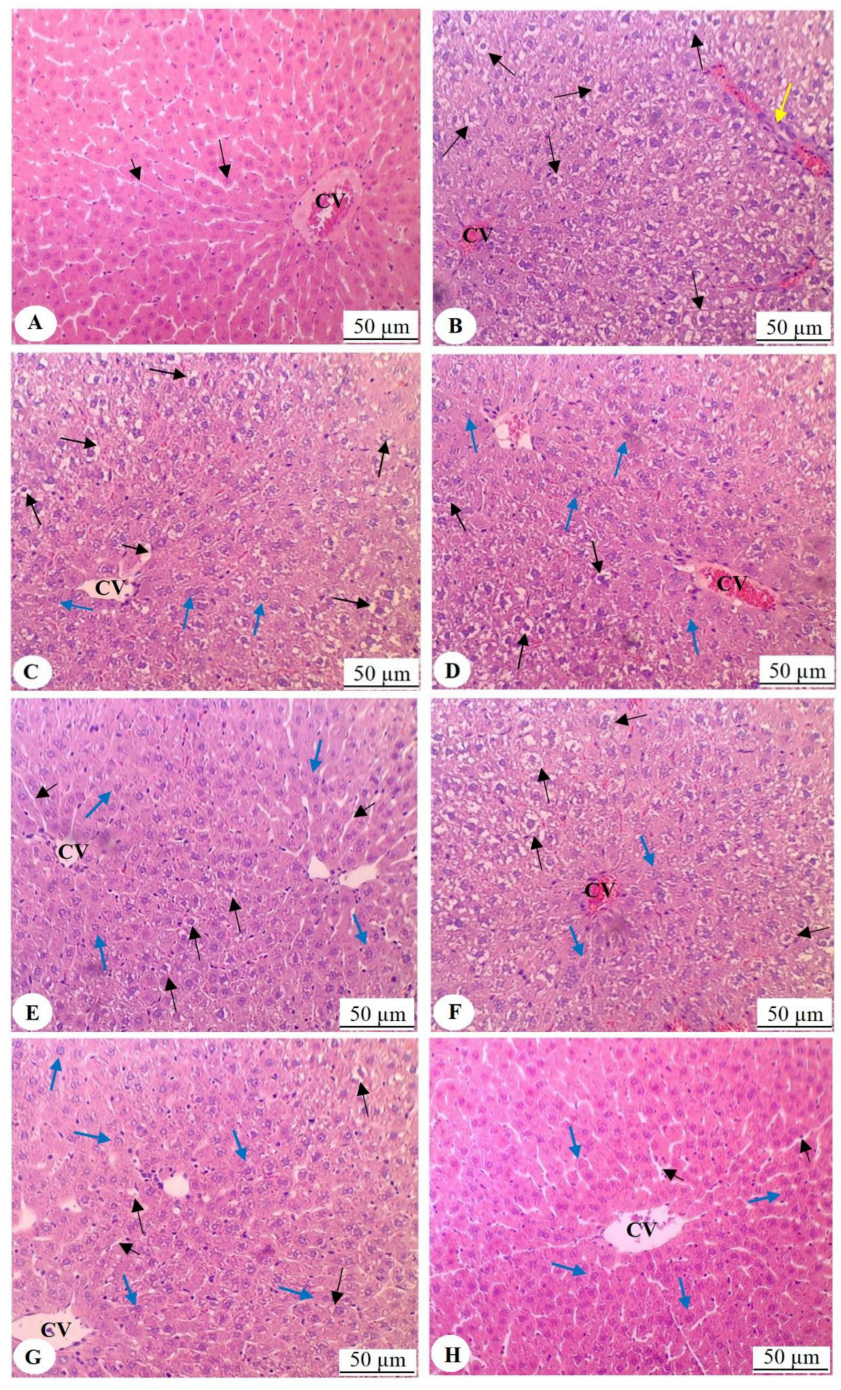
Histological analysis of liver tissues from all experimental groups, stained with hematoxylin and eosin and examined under light microscopy (200 × magnification). **(A)** Represents the liver of control rats, displaying normal histological architecture, including a well-structured and intact central vein (CV) with radially arranged oval cells emanating from the central vein (long black arrow). The sinusoids show normal size and morphology (short black arrow). **(B)** Illustrates the liver of rats fed a high-fat diet (HFD), where congestion of the central vein is observed, along with prominent cytoplasmic vacuolation in the majority of hepatocytes (long black arrow). This section also demonstrates increased infiltration of immune cells (yellow arrow). **(C)** Shows the liver of rats treated with HFD and Sidr honey (500 mg/kg), exhibiting a higher number of vacuolated hepatocytes (long black arrows) and fewer normal hepatocytes (blue arrows). The sinusoids remain of normal size around the central vein (short black arrow). **(D,E)** Show livers from rats treated with Sidr honey at doses of 700 mg/kg and 1000 mg/kg, respectively, with a progressive reduction in vacuolized hepatocytes (long black arrows) and a concomitant increase in normal hepatocytes (blue arrows). In these images, most cells appear normal. **(F)** Represents the liver of rats treated with HFD and Talh honey (500 mg/kg), where approximately half of the hepatocytes exhibit vacuolation (long black arrows), while the remaining hepatocytes appear normal (blue arrows). **(G)** Shows the liver of rats treated with HFD and Talh honey (700 mg/kg), where only a small number of hepatocytes display cytoplasmic vacuolation (long black arrows), with the majority of hepatocytes appearing normal (blue arrows). Normal-sized sinusoids are also evident (short black arrow). **(H)** Shows the liver of rats treated with HFD and Talh honey (1,000 mg/kg), where nearly all hepatocytes appear normal (blue arrows), and sinusoids exhibit normal morphology (short black arrows).

Hematoxylin and eosin staining of the liver tissues from the various groups of rats revealed distinct differences in their histology under a microscope. The liver in the control group (Figure 4A) looked normal and had intact central veins (CV) and oval cells arranged radially. The sinusoids were of normal size. In contrast, the HFD group had congested central vein, extensive vacuoles in hepatocytes’ cytoplasm, and increased immune cell infiltration (Figure 4B). Treatment with Sidr honey (500 mg/kg) (Figure 4C) resulted in a higher number of vacuolated hepatocytes, with fewer normal cells present. The sinusoids maintained normal size. Higher doses of Sidr honey (700 and 1,000 mg/kg) progressively reduced vacuolization. Simultaneously, the number of normal hepatocytes increased, and most of the cells appeared normal (Figures 4D, E). The Talh honey-treated group showed hepatocytes with numerous vacuoles occupying half of the field (Figure 4F), with normal cells occupying the other half. At 700 mg/kg (Figure 4G), the number of vacuolated cells decreased, the majority of hepatocytes appeared normal, and sinusoids had normal size. The 1,000 mg/kg dose of Talh honey (Figure 4H) resulted in nearly all hepatocytes appearing normal, with normal sinusoids observed throughout the liver tissue.

## 4 Discussion

Numerous studies have highlighted the potential of natural products and targeted dietary interventions in the prevention and management of MetS and its associated complications. Among these, various honey types have shown promising effects in mitigating MetS and obesity-related disorders (28, 37). In this context, the present study demonstrates the efficacy of Sidr and Talh honey from Saudi Arabia in attenuating obesity and NAFLD in HFD-fed rats, revealing distinct mechanistic differences. Sidr honey did not significantly impact body weight but effectively improved dyslipidemia and conferred hepatoprotection against steatosis and injury, likely via its hypoglycemic, antioxidant, anti-inflammatory, and anti-apoptotic actions. In contrast, Talh honey produced more substantial effects, including reductions in weight gain, hyperglycemia, and fat pad mass, along with inhibition of apoptosis and lipogenesis in hepatic and WAT tissues. Talh honey also markedly attenuated oxidative stress and inflammation, coinciding with nuclear translocation of Nrf2, contributing to its hepatoprotective profile. Interestingly, the data is showing that the mechanism of action of Talh honey alone involves activation of AMPK-mediated suppression of ACC, a central regulator of lipid metabolism, glucose handling, and inflammatory responses. On the other hand, Sidr honey failed to activate this pathway. Collectively, these findings support the potential role of Sidr and Talh honey as natural therapeutic agents for the management of obesity, IR, and NAFLD with different mechanisms of action.

In this study, we utilized only male Wistar albino rats, a common and scientifically justified practice in metabolic research. Male rodents were selected to minimize hormonal variability introduced by the estrous cycle, which can significantly influence fat distribution, insulin sensitivity, and hepatic lipid metabolism. Using males enhances the internal validity and reproducibility of the findings, particularly in high-fat diet models, where consistent metabolic responses are critical for evaluating intervention effects. While this design limits the ability to assess potential sex-specific responses, it allows for a more controlled assessment of honey’s metabolic effects and aligns with previous obesity studies that also used male models (57–60).

White adipose tissue (WAT) plays a critical role in systemic energy balance, glucose regulation, and lipid metabolism, serving both as a fat reservoir and an endocrine organ (61). In experimental science, the HFD model in rodent studies effectively simulates obesity, reflecting the effects of prolonged caloric surplus and consequent body weight increase, just as it does in humans (62, 63). However, the peripheral IR in the WAT and muscles plays a pivotal role in obesity and its systemic consequences. These cells become less responsive to insulin, which impairs glucose uptake and metabolism. This leads to hyperglycemia, WAT hypertrophy, and fat accumulation, all of which are major signs of obesity (64). In an interesting observation, Sidr honey did not significantly affect food intake, body weight, fat gain, insulin levels, or HOMA values as marker of IR in rats fed a high-fat diet (HFD). These data dissipate the any effects of Sidr honey on insulin release or action. It also suggests that Sidr honey has no impact on appetite, WAT lipid glucose, or lipid metabolism. Despite the limited number of studies specifically investigating Sidr honey from Saudi Arabia, other research supports these findings. For example, Mushtaq et al. (65) reported no impact of Alshifa Sidr honey on body weight in both normal and obese girls. Similarly, studies involving various honey types, such as wildflower forest thyme honey from Greece and an unidentified honey from Iran, also showed no effect on body weight in obese prepubertal girls or overweight adults (66, 67). However, Sidr honey did reduce fasting hyperglycemia in HFD rats, suggesting that it has hypoglycemic properties, though this effect appeared independent of changes in food intake or peripheral insulin resistance, pointing to alternative mechanisms of action. In fact, the independent hypoglycemic effect of various kinds of honey is well documented in healthy individuals, as well as in animal models and humans with types 1 and types 2 DM and has been reviewed extensively (68, 69). Several factors contribute to the hypoglycemic effects of honey, with fructose content being a key contributor. Fructose, which makes up between 21% and 43% of honey, has a low glycemic index of 19 (compared to glucose’s) (62, 70). Studies have shown that fructose lowers blood glucose by slowing its absorption in the intestines, delaying gastric emptying, and reducing food intake (39, 71–73). Additionally, fructose activates glucokinase in the liver, promoting the uptake and conversion of glucose into glycogen (74). Furthermore, some kinds of honey, such as the Trigona iridipennis honey, can reduce fasting glucose levels by inhibiting digestive enzymes like α-amylase and α-glucosidase (75). Moreover, other rat studies have shown that some honey phenols, such as quercetin, could alleviate hyperglycemia by stimulating insulin signaling and GLUT-4 expression through increasing the tyrosine phosphorylation on insulin receptor substrate (IR), and direct activation of the P13k/Akt pathway (39, 76, 77).

Excessive adipogenesis and lipogenesis in both WAT and the liver are key contributors to adipocyte hypertrophy and hyperlipidemia—hallmarks of metabolic syndrome. Adipogenesis occurs specifically in WAT, while lipogenesis affects both WAT and hepatic tissues, leading to fat accumulation (78). These processes are governed by a network of transcriptional regulators and enzymes, including SREBP-1, PPARs, and ACC, which modulate lipid metabolism by controlling downstream effectors such as FAS (79). Dysregulation of these pathways disrupts lipid homeostasis and is closely linked to obesity and related metabolic disorders (79, 80). AMPK, a critical energy sensor, suppresses lipogenesis and promotes fatty acid oxidation by phosphorylating and inhibiting ACC. In our study, WAT and liver tissues from HFD-fed rats showed reduced phosphorylation of AMPK and ACC, confirming impairment of this axis. These findings support the role of AMPK suppression in promoting lipogenesis, insulin resistance, hepatic steatosis, and NAFLD (21, 81–84), highlighting the importance of AMPK/ACC signaling in metabolic regulation.

Despite the high caloric density of honey, accumulating evidence suggests that various types, particularly those derived from *Acacia* species, exhibit significant anti-obesity, insulin-sensitizing, and hypolipidemic properties (85, 86). Notably, *Acacia* honey has been demonstrated to effectively reduce body weight as well as fasting glucose and insulin levels in rodent models (85, 87). Moreover, *Gelam*, *Acacia*, and *Geniotrigona thoracica* honeys have shown substantial efficacy in lowering serum triglycerides, total cholesterol, and LDL-c, while concurrently elevating HDL-c levels in obese and diabetic male rats (87, 88). These preclinical findings have been corroborated by several clinical trials in overweight and obese individuals, both with and without type 2 diabetes mellitus (T2DM) (66, 89–91), and further substantiated by systematic reviews (86, 92). The observed metabolic benefits of honey are largely attributed to its high phenolic content, which contributes to reduced adipocyte size and number, diminished lipid accumulation, and increased energy expenditure (66, 85, 87, 93). Nonetheless, the precise molecular mechanisms remain incompletely understood.

In the present study, both Sidr and Talh honeys exerted significant anti-hyperlipidemic effects, evidenced by dose-dependent reductions in serum and hepatic triglycerides, total cholesterol, and LDL-c levels. However, Talh honey demonstrated markedly superior efficacy across all doses. While Sidr honey produced a dose-dependent reduction in plasma glucose and HbA1c, Talh honey exerted broader and more pronounced metabolic improvements, including substantial reductions in fasting glucose, HbA1c, insulin levels, HOMA-IR, body weight, and adipose tissue mass. These results suggest that Talh honey may attenuate obesity and related metabolic disturbances through mechanisms that enhance peripheral insulin sensitivity—potentially by upregulating GLUT-4 expression and promoting glucose uptake in muscle and adipose tissues, while simultaneously suppressing hepatic and adipose lipogenesis and reducing serum free fatty acid levels. Importantly, only Talh honey significantly increased phosphorylation of ACC, likely via activation of AMPK in both hepatic and adipose tissues. This suggests that the activation of the AMPK/ACC axis underpins the potent hypoglycemic, hypolipidemic, and anti-obesity effects of Talh honey, consistent with AMPK’s established role in inhibiting lipogenesis and promoting insulin-stimulated glucose uptake (14). In contrast, Sidr honey failed to modulate the AMPK/ACC pathway, implying that its modest glycemic improvements are likely mediated through alternative mechanisms—possibly by inhibiting intestinal glucose absorption, suppressing hepatic gluconeogenesis, or reducing hepatic glucose output. Given the central role of AMPK in the pathogenesis and management of obesity, type 2 diabetes, and NAFLD (21, 94), these findings underscore the therapeutic potential of Talh honey, particularly *Acacia*-derived types, as promising natural modulators of metabolic health. Supporting this, *Manuka* honey has also been reported to activate AMPK signaling in dermal fibroblasts, further reinforcing the translational relevance of AMPK-targeting honey varieties (95).

Oxidative stress plays a crucial role in the pathophysiology of NAFLD, contributing to lipid peroxidation, IR, inflammation, fibrosis, and apoptosis (96). In conditions such as obesity, often driven by HFD consumption, excess caloric intake leads to the accumulation of free fatty acids in the liver, thereby exacerbating oxidative stress and triggering a cascade of metabolic disturbances. The accumulation of ROS impairs mitochondrial function, promotes IR, induces endoplasmic reticulum stress, and facilitates the infiltration of macrophages, all of which contribute to liver dysfunction (82, 97). In addition, these ROS-induced signaling pathways activate inflammatory cytokine cascades like NF-κB and create a self-reinforcing cycle by promoting fibrosis and apoptosis, which accelerates liver damage and the progression of NASH (97). Therefore, effective management of oxidative stress and inflammation is critical for mitigating the progression of NAFLD and other associated metabolic disorders. In the liver, a central defense mechanism against oxidative stress is Nrf2, a transcription factor that regulates the expression of antioxidant enzymes and other protective proteins (98). Researchers have found a link between Nrf2 disturbances in the liver and the development of NAFLD and other hepatic disorders. This highlights how important Nrf2 is as a target for therapy (99, 100). Under normal conditions, Nrf2 is bound to its inhibitor, kelch-like ECH-associated protein 1 (Keap1), in the cytoplasm. Upon oxidative stress, Nrf2 dissociates from Keap1 and translocates to the nucleus, where it activates the transcription of genes involved in the cellular antioxidant response. Notably, various signaling pathways, such as the phosphatidylinositol 3-kinase (PI3K)/Akt pathway, can also modulate the activity of Nrf2. A pathway that inhibits glycogen synthase kinase 3 beta (GSK-3β), which is a Nrf2 negative regulator (79). Additionally, AMP-activated protein kinase (AMPK), which is a key regulator of cellular energy balance, has also been shown to activate Nrf2 either by phosphorylating it directly or by inhibiting GSK-3β, thereby enhancing its antioxidant response (101).

In the current study, hepatic tissues from HFD-fed rats exhibited markedly reduced levels of both Nrf2 mRNA and nuclear Nrf2 protein, indicating impaired transcriptional expression and nuclear transactivation. This deficiency was associated with elevated hepatic levels of MDA, TNF-α, and IL-6, alongside reduced antioxidant defenses, including SOD, GPx, and HO-1. Such dysregulation suggests that Nrf2 translocation may be compromised through both Keap1-dependent and independent mechanisms, leading to weakened antioxidant responses and heightened oxidative stress. Furthermore, increased expression of pro-apoptotic markers—Bax, cytosolic cytochrome-c, and caspase-3—reflects ongoing cellular injury and apoptosis within the liver.

Despite comparable food intake among all experimental groups, only Talh honey elicited a significant, dose-dependent reduction in body weight, while Sidr honey had no effect on weight gain, neither attenuating nor exacerbating it. This observation initially raises a paradox: both types of honey are rich in simple sugars and thus expected to contribute additional calories, yet Talh honey mitigated weight gain, and Sidr honey maintained body weight at levels similar to the HFD group. These findings are consistent with previous studies demonstrating that honey-fed animals often exhibit less weight gain than those receiving equivalent amounts of refined sugars, such as sucrose or glucose, despite similar or higher caloric intake (102, 103). Mechanistically, honey’s diverse polyphenolic content, including flavonoids such as quercetin and kaempferol, has been shown to enhance energy metabolism, increase thermogenesis, delay gastric emptying, and reduce intestinal absorption, factors that collectively reduce net energy gain (33, 104, 105). Moreover, Ladas et al. (106) demonstrated that honey can exert mild laxative effects and improve gastrointestinal motility, which may further explain reduced energy efficiency in honey-fed animals. In our study, Talh honey’s ability to activate the AMPK/ACC signaling axis in adipose tissue likely plays a central role in its anti-obesity effects. AMPK activation suppresses adipogenesis and lipogenesis while promoting fatty acid oxidation and energy expenditure, ultimately resulting in weight reduction independent of caloric intake. In contrast, Sidr honey, although not activating the AMPK/ACC pathway, prevented additional weight gain compared to the HFD group. This suggests that Sidr honey may help stabilize weight through other mechanisms, potentially via modulation of glycemia, attenuation of oxidative stress, or improved insulin sensitivity, without exacerbating adiposity, despite its sugar content. The relatively lower total phenolic content of Sidr compared to Talh may account for the absence of a weight-reducing effect, but its bioactive profile may still suffice to offset the obesogenic impact of a high-fat diet. Taken together, these findings reinforce the concept that not all caloric sources have equal metabolic consequences. Honey’s complex matrix of phytochemicals appears to modulate energy balance through mechanisms that transcend simple calorie counts, particularly in the case of Talh honey, which demonstrated potent anti-obesity efficacy in this model.

Interestingly, both Sidr and Talh honey attenuated the oxidative and inflammatory milieu in HFD rats by reducing MDA, pro-inflammatory cytokines, and apoptotic markers, supporting their role as natural antioxidants. However, Talh honey demonstrated superior antioxidant efficacy compared to Sidr, consistent with previous reports on the high antioxidant potential of honeys rich in flavonoids, enzymes (e.g., catalase, GOX, SDH, ACP), and vitamins (e.g., Vit C, B6, riboflavin, folate, niacin) (38, 107). Importantly, only Talh honey significantly increased nuclear Nrf2 protein levels, without altering Nrf2 mRNA, suggesting post-transcriptional activation of the Nrf2 pathway. No such effect on the expression or activation of Nrf2 were observed with Sidr honey. This finding implies that Talh exerts its antioxidant effects at least partly via activation of the Nrf2 axis, possibly through stimulation of hepatic AMPK. AMPK is known to enhance Nrf2 nuclear translocation and transcriptional activity through multiple mechanisms (101). This interaction is further supported by prior evidence of AMPK/Nrf2 pathway activation by Manuka honey in oxidative stress models (95). In contrast, the antioxidant and anti-inflammatory effects of Sidr honey may be mediated by alternative pathways, such as direct ROS scavenging or inhibition of hepatic ROS production via its hypoglycemic and anti-hyperlipidemic effects as conformed form this study.

It’s still not clear exactly what the mechanism of action is for how honey combats obesity and protects the liver from damage, but some studies have linked these benefits to its high phenolic content, which includes flavonoids that protect against diabetes and liver damage (28). In previous research, the compositional analysis of both Talh and Sidr honey reveals complex biochemical matrices that explain their distinct bioactivities observed in this study. Sidr honey is particularly rich in carbohydrates (76.1–79.2 g/100 g) and exhibits high levels of total phenolics (up to 89.3 mg GAE/100 g) and flavonoids (18.2 mg CE/100 g), with major compounds including quercetin, kaempferol, rutin, and phenolic acids such as caffeic acid (108, 109). GC-MS analysis identified volatiles like methyl benzaldehyde and some essential minerals (K, Ca, P) (108). On the other hand, Talh honey is lower in total phenolics (∼54 mg GAE/100 g) but is very rich in unique and potent compounds such as benzoic acid, gallic acid, vanillic acid, and quinic acid, especially concentrated in its water and ethyl acetate extracts (110). Its antioxidant activity, measured through DPPH and ABTS assays, is significantly enhanced after fractionation, notably exceeding that of Sidr honey in multiple extracts (110). These compositional differences provide mechanistic insight into the superior metabolic outcomes elicited by Talh honey in HFD-fed rats. While both honeys attenuated hepatic injury via antioxidant and anti-apoptotic mechanisms, this effect could be attributed to their free radical scavenging and antioxidant potential. However, Talh honey significantly reduced body weight, adiposity, and hepatic lipogenesis. This is likely due to its activation of the AMPK/Nrf2 axis—a key pathway in energy homeostasis and lipid metabolism. These effects could be attributed to its high content of gallic acid, vanillic acid, and quinic acid. Indeed, gallic acid has been shown to reduce body weight, improve glucose handling, and suppress hepatic lipogenesis and inflammation via AMPK activation (111, 112). Also, vanillic acid enhances AMPK signaling and thermogenic activity in the liver and adipose tissue (113, 114). In the same manner, when given as a food supplement to mice, D-(–)-quinic acid attenuated dyslipidemia and suppressed adipogenesis and body weight gain by activating AMPK (115). In addition, the higher free acidity and lower pH of Talh honey compared to Sidr honey may serve as another physiological activator of AMPK (116). Acidic stress is a recognized inducer of AMPK activation, particularly in ischemic tissues and tumor cells adapted to low pH (117–119). This acid-mediated AMPK activation may complement the flavonoid-induced signaling to produce a stronger metabolic response. Thus, while Sidr honey exhibited antioxidant and hepatoprotective effects, likely due to its high phenolic load, Talh honey’s broader efficacy against obesity, hyperglycemia, and NAFLD stems from its unique phytochemical profile, including gallic acid, vanillic acid, quinic acid—and its physicochemical properties that jointly activate AMPK and Nrf2 pathways more robustly.

## 5 Conclusion

Sidr and Talh honey from Saudi Arabia demonstrate significant potential in mitigating obesity and liver damage induced by HFD in rats, although their mechanisms of action differ. Sidr honey primarily exerts hypoglycemic, antioxidant, and anti-inflammatory effects, protecting the liver and improving metabolic parameters without significantly affecting body weight or on AMPK, ACC, and Nrf2 activation. In contrast, Talh honey, derived from acacia, exhibits more significant effects such as reducing body weight, hyperglycemia, and hepatic steatosis. This most likely occurs through the activation of AMPK and Nrf2 signaling pathways in both liver and white adipose tissue. The higher phenolic content in Talh honey likely contributes to its superior antioxidant and anti-obesity effects. Future studies should focus on identifying the specific bioactive compounds responsible for these effects and further elucidating their molecular mechanisms to optimize their therapeutic potential for metabolic disorders like obesity and NAFLD.

### 5.1 Study limitations

This study has several limitations that should be considered when interpreting the results. Due to the distinctive physical properties of honey, blinding during its administration was not feasible; however, outcome assessments were conducted by investigators blinded to group allocations to minimize bias. Only male rats were used, which limits the generalizability of the findings to females, given the well-established influence of sex hormones on obesity and metabolic responses. While the study demonstrated significant metabolic benefits of Sidr and Talh honey, we did not identify the specific bioactive compounds responsible, nor did we employ pathway inhibition or knockout models to confirm the mechanistic roles of AMPK and Nrf2, making the mechanistic conclusions correlative rather than causal. The study duration was relatively short, preventing evaluation of long-term outcomes, and the lack of human clinical data restricts translational relevance. Moreover, the absence of an isocaloric or sugar-matched control group limits our ability to determine whether the observed effects are attributable solely to honey’s bioactive profile rather than its caloric contribution. The honey used, although well-characterized, may vary in composition due to geographic and botanical differences, which could affect reproducibility across different contexts. Additionally, while liver histology was examined by a blinded pathologist, the analysis remained qualitative, and no semi-quantitative scoring system was applied. Future studies should include validated histological scoring methods or digital image analysis to enhance objectivity. Although the Sidr and Talh honeys were obtained from certified suppliers and labeled as unifloral, we did not perform palynological (pollen) analysis to confirm their botanical origin. Therefore, classification as unifloral honey cannot be conclusively verified according to WHO/FAO or International Honey Commission standards, and this should be addressed in future studies. Lastly, the relatively small sample size and absence of comparison to conventional pharmacological treatments limit both the statistical power and the assessment of honey’s relative efficacy. Addressing these limitations in future research would enhance the robustness and translational value of the findings.

## Data Availability

The datasets presented in this study can be found in online repositories. The names of the repository/repositories and accession number(s) can be found in the article/supplementary material.
